# Animal welfare incidents during and after transport to Australian export slaughterhouses: An evaluation of government reports (2020–2021)

**DOI:** 10.1002/vro2.70009

**Published:** 2025-05-05

**Authors:** Francesca Carnovale, David R. Arney, Dawn Lowe, Clive J. C. Phillips

**Affiliations:** ^1^ Animal Nutrition, Institute of Veterinary Medicine and Animal Sciences Estonian University of Life Sciences Tartu Estonia; ^2^ Animals' Angels Frankfurt am Main Germany; ^3^ Curtin University Sustainability Policy (CUSP) Institute Curtin University Perth Western Australia Australia

**Keywords:** animal welfare incident reports, Australia, slaughterhouse

## Abstract

**Background:**

Australian animals delivered to meat export slaughterhouses are inspected ante‐mortem for welfare problems by a veterinarian, which determines their fitness to enter the slaughter process. Emergency killing, priority slaughter or proceeding to slaughter with other animals are possible outcomes. However, when animals with welfare conditions are detected, the veterinarian or slaughterhouse staff are required to send an incident report to the State government authorities.

**Methods:**

We reviewed 631 incident reports from 2020/2021, obtained from the Australian government following Parliamentary Enquiry.

**Results:**

Emergency killing was the most common response to incidents, especially after long journeys. Pigs had the most incidents, mostly handling issues not requiring emergency killing. Cattle had the second highest and were the subject of the most reports. Lameness was commonly recorded. Delivery to detection took 8–12 hours on average, probably because deliveries late in the day are inspected the following day.

**Conclusions:**

Incident reports identified that there were significant welfare problems with farmed animals arriving at meat export slaughterhouses, especially in pigs. Lengthy delays to detection were also identified. Reducing this time to detection would improve welfare, as would ensuring slaughterhouses have a system to monitor all animals at all times.

## INTRODUCTION

Good farmed animal welfare is only possible if standards are effectively maintained during all periods of the animals’ lives, including before, during and after transport.^1^ Australia has some of the most remote cattle and sheep farms, which are a long way from markets and slaughterhouses, and it transports farmed animals for some of the longest distances in the world.^2^

Slaughtering of farmed animals is conditional upon there being no threat to the safety of the meat product, as determined by an on‐plant veterinarian (OPV). Under Australian Standard (AS) 4696:2023, farme animals suspected of illness or injury are referred to the OPV for approval, or otherwise, to progress through the slaughter process.^3^

If an animal does not comply with the AS due to an animal welfare issue, an Animal Welfare Incident Report (AWIR) should be initiated by staff at the receiving slaughterhouse or by the OPV. Slaughterhouse management has the legal responsibility to ensure that animals are killed with minimum harm and distress to the animal.^4^

If there is a suspected animal welfare violation in an export slaughterhouse, and an AWIR is completed, this is sent to the relevant State or Territory regulatory authority (State and Territory governments are responsible for animal welfare), which decides whether to investigate the incident. The Australian federal government is responsible for ante‐mortem inspection of animals, post‐mortem inspection procedures, overseeing animal welfare and checking that all company activities comply with relevant legislation (https://www.agriculture.gov.au/sites/default/files/sitecollectiondocuments/animal‐plant‐health/animal/the_office_of_the_chief_veterinary_officer/vet‐brochure.pdf). The OPV has oversight of animal welfare but no powers to investigate and/or prosecute.

If non‐compliance is discovered at a facility and the OPV issues a corrective action request (CAR) to the facility, the facility must correct this. If left uncorrected, the regulatory authority has the power to impose sanctions, which can include revoking the facility's license.

To avoid non‐compliance with transport regulations, procedures have been developed to prevent the transport of welfare‐compromised animals, directing farmers to undertake on‐farm emergency slaughter (OFES). This is also the case in the UK, Australia, New Zealand and Canada.^5^, ^6^, ^7^ The Farm Animal Welfare Advisory Council (FAWAC) in the Republic of Ireland has published Animal Welfare Guidelines for the Management of Acutely Injured Animals on Farm.^5^, ^6^, ^7^ However, even though these procedures exist, animals may still be transported in an unfit state.^8^ The latest UK government Annual Animal Welfare Report, for 2023/24^8^, shows that non‐compliant transport and farm practices in the UK reduced by 8% compared with 2022/2023.^8^ However, in slaughterhouses, there has been little change in the number of non‐compliant cases over the past 3 years.^8^ The UK government aims to reduce the number of non‐compliances requiring emergency kills, which by their definition require immediate action.^9^ Likewise, EU Council Regulation (EC) No 1099/2009 aims to reduce the suffering of farmed animals at killing. Inspection must take place within 24 hours of arrival at the slaughterhouse and less than 24 hours before slaughter.^10^ Where animals have experienced pain or suffering during transport, they must be slaughtered immediately following arrival,^11^ with the FBO (food business operator) being responsible.

UK National surveys show average waiting times of just 23 minutes before unloading at slaughterhouses, but individual vehicles sometimes wait for 3–5 hours.^12^ If the waiting time exceeds 30 minutes, it is the responsibility of the official veterinary surgeon (OVS) to take action. The OVS must be present during unloading and cooperate with local authorities on transport issues. Local authorities must check animal transporting vehicles regularly, ensuring sufficient space for unloaded animals at the destination. Where an animal is in obvious pain or distress, it must be killed without delay rather than waiting for an antemortem inspection.^13^, ^14^

In the EU, following on‐farm killing, meat may enter the food chain if deemed suitable. However, many slaughterhouses do not support this.^6^, ^15^ At the end of 2023, the European Commission committed to revising animal welfare legislation, especially concerning transportation and time of killing. There are many cases of non‐compliance with farme animal welfare legislation in the EU, both during transport and on‐farm.^16^ Sanctions may be invoked when someone transports unfit animals, or there is limited feed and space during transportation. Concerning non‐compliance at slaughterhouses, information on the outcome of official animal welfare controls is not available in eight of 27 EU countries. Of countries that detailed non‐compliance procedures, none provided reasons for non‐compliance.^16^ Measures to improve animal welfare include increasing inspections, detailed reporting of non‐compliance and detecting unfit animals on farms, not on arrival at slaughterhouses.^17^ However, EU Member States still need to ensure that slaughterhouse checks are made by competent authorities. The number of inspections required per year is not specified in EU regulations.^18^ There is evidence of problems with inspections and emergency kills on farms in several EU countries,^15^ including high levels of non‐compliance.^19^, ^20^, ^21^ The importance of training to detect animal welfare problems before animals are transported and encouragement of good decision‐making is emphasised.^22^ Farmers have a positive opinion towards the use of OFES,^23^, ^24^ but this procedure offers little economic benefit and is not used due to a lack of slaughterhouses offering this service. In Canada, transportation of unfit animals is also a common cause of non‐compliance with Health of Animal Regulations.^25^, ^26^

Ways of reducing animal suffering during transportation, including detection of lame animals on farms, making transportation payments based on the condition of animals at delivery, not paying drivers based on the weight of livestock transported, avoiding overloading of animals and providing rewards to the slaughterhouse if there is less bruising or fewer dead animals arriving at the slaughterhouse have been advocated.^27^ In Australia, 0.005% of the 36 million animals sent to red meat export establishments in 2021 were included in an AWIR, a total of 278 reports.^28^ Even though there were COVID‐related restrictions during this period, trade in animals continued and, to the best of the authors' knowledge, those responsible ensured there was no interruption of welfare checks and they were carried out correctly. By contrast, the UK food transport industry lost staff in all logistic chains during the COVID pandemic.^29^

We investigated the information available from AWIRs, and National Vendor Declarations (NVD), which were attached to each AWIR, to determine the prevalence of conditions reported at Australian export slaughterhouses, the timing of reporting and the actions taken.

## METHODS

### Data collection

The data used in this study were curated and synthesised from two sources, AWIRs and NVDs (Box S1). The NVD is a legal document for traceability, which details information on the food safety status of animals being sold into the food chain. The NVD should be completed and provided by farmers, feedlotters or saleyard (market) staff to the driver when the consignment (a group of animals from a single source, sent on a single vehicle) leaves the farm, feedlot or saleyard. Drivers may collect several groups of animals and so have different ‘consignments’ and thus several NVDs on one truck. The information includes the animals' owner, the establishment address, the number and sex/type of animals, health information, the time the transport commenced and their destination.

The AWIR contains 11 sections that the person(s) declaring an animal welfare incident must complete. Section A details the background and purpose of the report. Section B details the incident description, date, detection time, arrival time, unloading time, animal count, nature of the incident, reasons for unfit transport, the affected animal species, class, sex, breed, age, ear tag, brand, tattoos, BCS (body condition score), behaviour, owner and property origin information, saleyard (market) origin (if applicable) and sale date. Section C details the transportation, including delays or lay‐overs, stops during the trip, availability of water, head clearance, condition of the truck, journey duration and stocking density. Sections D, E and F provide space to include photographs of the incident, the action taken to alleviate animals’ suffering and who reported the incident.

The AWIRs used in our study were released through an Australian Parliamentary inquiry and made available by the Parliament of Australia.^28^, ^30^, ^31^ There were 631 AWIRs registered at slaughterhouses between 1 January 2020 and 31 December 2021, although 64 were withheld for state and territory investigations. The reports provide a comprehensive description of incidents involving animals delivered to meat export slaughterhouses and, where relevant, saleyards.

### Dataset structure

**FIGURE 1:**
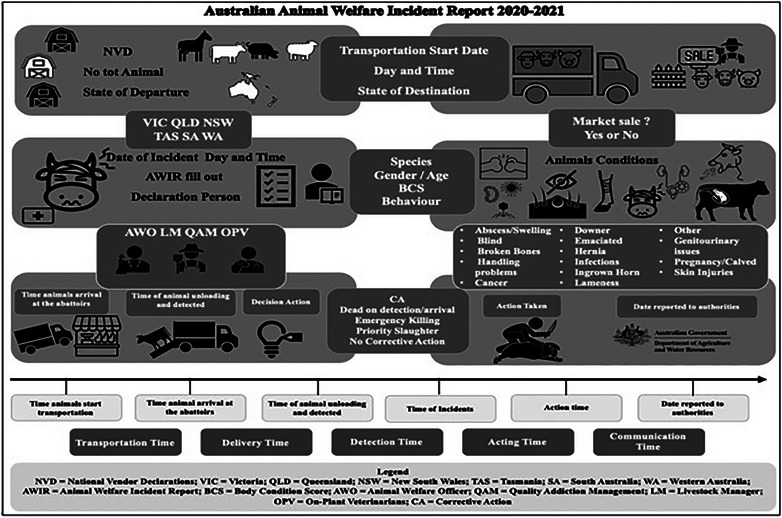
Explanatory diagram of Animal Welfare Incident Reports.

From the 567 AWIR, and accompanying NVDs where provided, the following information was recorded: total and affected number of animals in incidents sustained during transport, number of incidents to date for that slaughterhouse that year, species (cattle, pigs, sheep or horses), gender (female, entire male or neutered male), age, departure and arrival locations, BCS (poor, acceptable or good), behaviour (normal/calm, mildly stressed or severely stressed/distressed), timestamps for transport and action‐related events, Corrective Action decisions (emergency killing [EK], priority slaughter [PS], no corrective action [NCA], or dead on detection/arrival [DoD/A]) and identification of the responsible party for the declaration (veterinarian or manager) (Table 1, Figure 1).

**TABLE 1:** Definitions of mitigating corrective action (CA) decisions and action‐related events for animals arriving at slaughterhouses; CAs are from Australian Standards (2007).^3^

	Definitions
***Corrective action decision***	
Emergency killing	Killing by necessity of any animal that has recently suffered traumatic injury or is affected or suspected of being affected by disease or other abnormality or will deteriorate unless it is killed immediately. Animals are usually moribund and killed in situ
Priority slaughter	Killing of any animal that is in pain or is likely to deteriorate unless killed immediately. The flow of animals on the kill floor is interrupted to facilitate this. Animals can walk or be forced to walk to the kill floor
No corrective action	Animals are not in pain and there is no necessity to kill them immediately. Animals proceed through the slaughter process normally with the rest of the consignment or are returned to owner
**Action‐related events**	
Transportation time	Duration from the start of transportation to delivery, hours
Delivery–Action	The time between animal delivery and the execution of necessary actions, if any, hours
Delivery–Detection	Time elapsed between animal delivery and the incident identification, hours
Detection–Action	Time elapsed between incident identification and the subsequent implementation of corrective measures, if any, hours
Detection–Communication	Time elapsed from incident detection to declaration by the vendor, days
Action–Communication	Time elapsed from the action taken to declaration by the vendor, days

*Note*: In the standards, emergency killing is referred to as emergency slaughter,^1^, ^3^ but as the animals are not necessarily used for meat, we have used the term ‘killing’.

**FIGURE 2:**
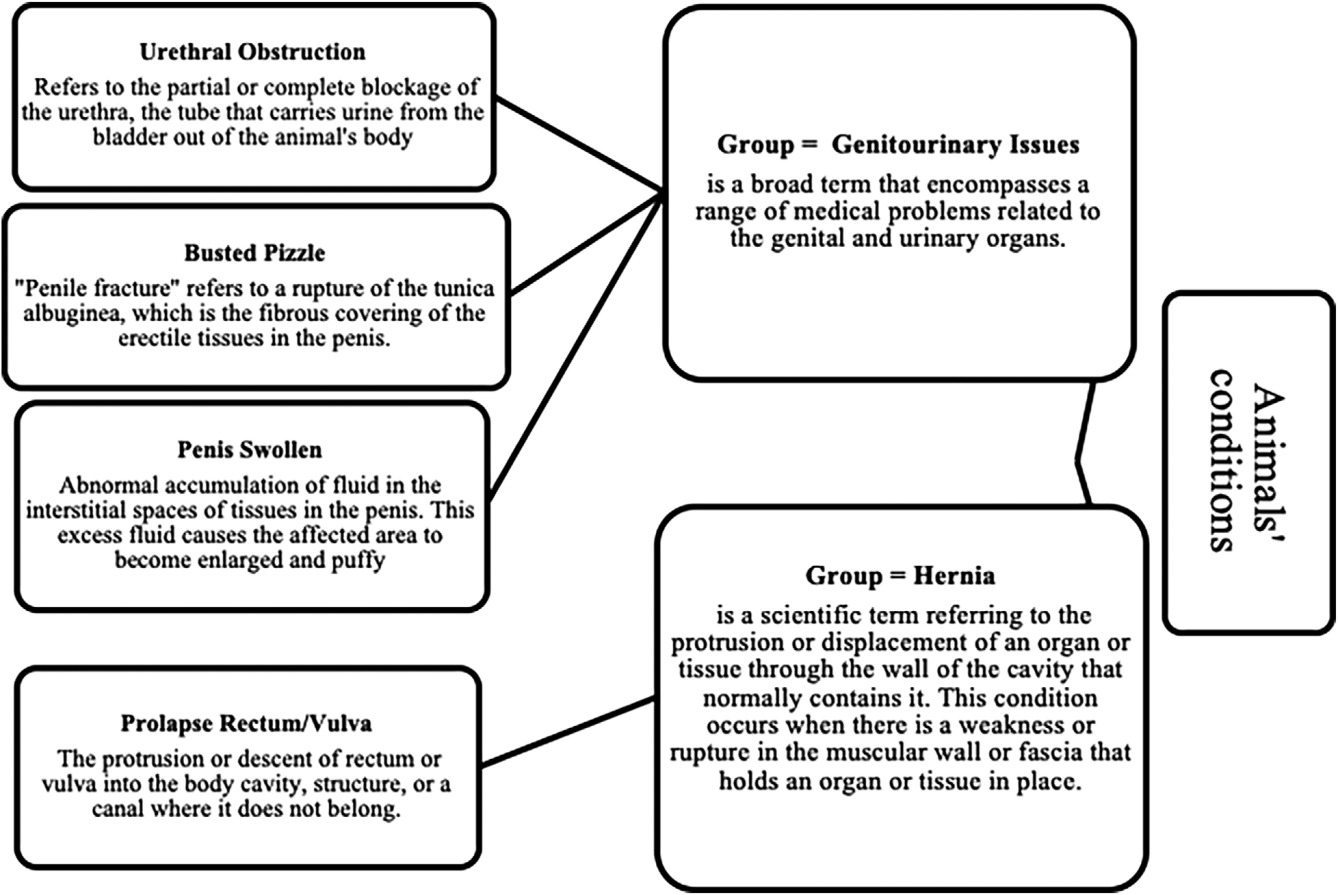
Example of definitions of similar conditions from the original reports that were incorporated into groups.

**FIGURE 3:**
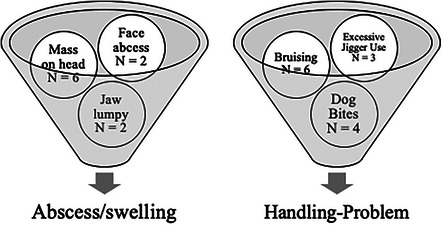
Example of conditions detailed in the reports that were amalgamated.

To promote robust statistical analysis, data with a small number of occurrences were stratified and grouped for similar classes and conditions (Table 2). The conditions of animals declared in the original reports were usually completed manually without standard definitions to describe the state of the animals. To collect and prepare the animal conditions for statistical analysis, definitions were synthesised and categorised to amalgamate similar conditions (Figure 2). In some cases, the number of animals affected was too small to create a condition group, and incidents were incorporated into a group of other conditions (Figure 3). The final categories of conditions were: abscess/swelling, blindness, broken‐bones, cancer, death, downer, handling problems, infections, ingrown‐horn, injuries, lameness, pregnancy/calved, skin injuries, emaciated, genitourinary‐issues, hernia and other conditions.

**TABLE 2:** Definitions of groups of conditions (numbers affected were less than 10) and number of affected animals.

Final group for statistical analysis	Conditions (bold = amalgamated groups)	Total number of reports	The total number of animals affected
**Abscess/Swelling**	Jaw abscess		
	Jaw lumpy		
	Mass on neck		
	Face abscess		
	Head swelling—massive		
	Mass on head		
	Mass on the eye with pus		
	**Abscess/Swelling**	**13**	**17**
**Blind**	**Blind**	**53**	**87**
**Broken bones**	Broken leg		
	Broken ribs		
	Broken bones		
	**Broken bones**	**33**	**48**
Cancer	Eye cancer		
	Vulva cancer		
	Mouth cancer		
	Cancer with maggots		
	Tail cancer		
	Cancer		
	**Cancer**	**49**	**80**
**Dead**	**Dead**	**32**	**140**
**Downer**	**Downer**	**36**	**201**
**Handling problems**	Bruising		
	Excessive jigger use		
	Driver handling		
	Dog bites		
	Dogs unmuzzled		
	**Handling problems**	**19**	**1180**
**Infections**	Infections		
**Infections**	Mastitis		
	**Infections**	**11**	**160**
**Ingrown horn**	**Ingrown horn**	**109**	**126**
**Injuries**	Injuries		
	Injury to tail		
	Injury lip		
	Injury large		
	Laceration		
	Injury abdomen		
	Injury to vulva and leg		
	Ear injuries		
	Back injuries		
	Eye injuries		
	Legs injuries		
	**Injuries**	**29**	**136**
**Lameness**	**Lameness**	**120**	**671**
**Pregnant/calved**	Pregnant/Calved		
	Pregnant		
	**Pregnant/Calved**	**26**	**846**
**Skin injuries**	Skin injuries		
	Flystrike		
	Shot/Arrow		
	Lesions		
	Sunburn		
	Heat Stress		
	**Skin injuries**	**17**	**201**
**Emaciated**	**Emaciated**	**13**	**82**
**Genitourinary issues**	Urethral obstruction		
	Penis problems		
	Pizzle bleeding		
	Pizzle swollen		
	Penis prolapsed		
	Busted pizzle		
	**Genitourinary issues**	**14**	**32**
	Prolapsed rectum/vulva	8	18
**Hernia**	Hernias—scrotal or umbilical		
	Hernia abdomen		
	Hernia and septic peritonitis		
	**Hernia**	**14**	**34**
**Other**	Distressed		
	Smothering in kill race		
	Tail rotting		
	Untipped horns		
	Kill procedure		
	Vulva with ear tags		
	Suffocated at slaughterhouse		
	Calcium deficiency		
	**Other**	**15**	**106**

Defined time‐related metrics regarding transport and mitigation procedures were identified from their anticipated relationships with animal responses during transportation (e.g., 4–7) (Table 2). Unrealistic time entries, such as those resulting in negative time values, were assumed to result from mistyping and were corrected or discarded if this was not possible.

### Statistical analysis

Statistical analyses were conducted using R version 4.3.0 (R Foundation for Statistical Computing). Spearman's correlation coefficients (*r*) from the raw and untransformed data described relationships between pairs of variables, using both the overall dataset and each species. Following this, principal component analysis (PCA) assessed relationships between multiple variables, identifying redundant descriptors and facilitating interpretation.^32^ Variables were scaled to zero mean values and unit variance to avoid the influence of measurement units on each variable's contribution to the dataset.

## RESULTS

### Descriptive statistics

Of the 567 AWIRs, 367 were related to cattle, 158 to sheep, 35 to pigs and seven to horses (Table 3). Considering the numbers of transported animals on affected consignments, sheep were the most numerous (63,883), then cattle (25,486). Similarly, sheep were the most affected animals, and then cattle and pigs. There were only seven horses affected. For sheep, there were more females than males, but pigs and cattle had almost equal gender representation, and in cattle most males were entire. Ewes were the oldest, then bulls and then wethers. The most common condition was handling problems, mostly because of the driver, but also because of unmuzzled dogs and dog‐bites (Table 2). The second most common condition was pregnancy/calving; then, in order of declining prevalence: lameness, skin injuries, downers, other‐injuries, ingrowing‐horns, dead, broken‐bones, blind, emaciated and cancer. Conditions depended on species, except handling problems, which were prevalent across all species, but particularly common in pigs (66.4%) (Table 3). Horses were not affected by handling problems. For cattle, the next most common conditions after handling problems were lameness and injuries. For sheep, lameness was most prevalent, and for pigs, most had handling problems, infections and skin injuries. Most of the affected horses were lame. BCS in the cattle was reported to be poor, for sheep, it was acceptable, and pigs and horses were reported to have good BCS. Cattle were assessed as mildly distressed, while sheep were generally calm. Pigs and horses were the most distressed species. More pigs were NCA compared to other species, mostly EK, with an intermediate number of PS. Sheep were the most likely to arrive dead.

**TABLE 3:** Number of reports, transported animals and affected animals of different types, conditions and corrective actions for cattle, sheep, pigs and horses.

Species	All animals^*^	Cattle	Sheep	Pigs	Horses
Number of reports (no.)	571	367	162	35	7
Transported animals (no.)	97,248	25,486	63,885	7694	183
**Affected animals (no.)**	**4304**	**1169**	**2245**	**883**	**7**
**Gender % of those affected (and no.)**					
Female	59.1 (2273)	44.8 (565)	69.7 (1452)	51.5 (252)	57.1 (4)
Entire male	36.4 (1397)	43.7 (550)	29.1 (607)	48.5 (237)	42.9 (3)
Neutered male	4.5 (171)	11.5 (146)	1.2 (25)	‐	‐
**Mean age (months)**					
Female	23.7	22.3	63.7	5.5	132
Entire male	33.7	36.7	12.7	5.5	72
Neutered male	11.2	9.3	25.8	‐	‐
**Conditions % of affected and (no.)**					
Abscess/Swelling	0.4 (17)	0.8 (9)	0.4 (8)	‐	‐
Blind	2.2 (87)	6.8 (77)	0.4 (8)	0.2 (2)	‐
Broken bones	1.2 (48)	2.2 (25)	1.0 (20)	0.3 (3)	‐
Cancer	2.0 (80)	1.6 (18)	3.1 (62)	‐	‐
Downer	5.1 (201)	3.0 (34)	8.0 (159)	0.9 (8)	‐
Emaciated	2.0 (82)	2.6 (30)	2.6 (52)	‐	‐
Handling problems	30.0 (1180)	33.9 (375)	11.2 (221)	66.4 (584)	
Hernia	0.8 (34)	0.3 (3)	0.3 (5)	3.0 (26)	‐
Infections	4.0 (160)	5.5 (63)	0.2 (4)	10.6 (93)	‐
Ingrown horn	3.2 (126)	9.3 (106)	1.0 (20)	‐	‐
Injuries	3.5 (136)	11.7 (122)	0.6 (11)	0.3 (2)	16.7 (1)
Lameness	16.0 (671)	16.2 (185)	23.9 (473)	0.9 (8)	83.3 (5)
Other	2.6 (106)	4.8 (56)	1.3 (27)	2.6 (23)	‐
Genitourinary issues	0.8 (32)	1.3 (15)	0.9 (17)	‐	‐
Pregnancy/Parturition (calved)	21.1 (846)	1.7 (20)	42.7 (825)	0.1 (1)	‐
Skin injuries	5.0 (201)	0.5 (6)	3.3 (66)	14.7 (129)	‐
**BCS, % of affected and (no.)**					
Poor = 1	13.0 (470)	43.8 (413)	2.8 (52)	0.9 (5)	0
Acceptable = 2	50.0 (1665)	20.2 (190)	75.3 (1424)	9.3 (51)	0
Good = 3	37.0 (1256)	36.0 (339)	21.9 (416)	89.8 (494)	100 (7)
**Behaviours, % of affected and (no.)**					
Normal/Calm	51.9 (1670)	24.6 (224)	74.7 (1348)	19.1 (95)	42.9 (3)
Mildly stressed	21.8 (699)	55.2 (502)	8.1 (146)	10.0 (50)	14.2 (1)
Severely stressed/distressed	26.4 (850)	20.2 (184)	17.2 (310)	70.9 (353)	42.9 (3)
**Corrective action decisions, % of affected and (no.)**					
No corrective action	18.4 (753)	6.5 (76)	4.4 (93)	68.0 (583)	16.6 (1)
Priority slaughter	17.8 (732)	11.2 (130)	27.3 (564)	4.5 (38)	‐
Emergency killing	60.4 (2476)	79.4 (927)	63.4 (1312)	27.0 (232)	83.4 (5)
Dead on detection/arrival	3.4 (140)	2.9 (34)	4.9 (102)	0.5 (4)	‐

Median transportation (transit) time was 4.7 hours and this was longer for horses than pigs, with cattle and sheep intermediate (Table 4). Median delivery to detection time was 10.8 hours and detection to action 0.5 hour, shorter for sheep and pigs than for cattle and horses. Median action to communication time was 1 day, which was the shortest for sheep.

**TABLE 4:** Duration of transportation, delivery to detection, detection to action and action to communication for cattle, sheep, pigs and horses.

Variable	Transportation time (hours)	Delivery–detection time (hours)	Detection–action time (hours)	Action–communication time (days)
**All animals**				
Median	4.7	10.8	0.5	1
Range	^a^–23.6	0–23.8	0–23.5	0–72
**Cattle**				
Median	5.0	11.8	0.8	1
Range	^a^–23.6	0–23.9	0–23.5	0–72
**Sheep**				
Median	5.1	7.83	0.17	0
Range	^a^–22.7	0–23.8	0–23.5	0–45
**Pigs**				
Median	3.5	8.1	0.2	1
Range	1.2–21.0	0–23.8	0–15.5	0–9
**Horses**				
Median	7.0	11.0	1.1	1
Range	0.5–11	1.5–14.6	0.1–21.0	0–3

^a^0 or unrecorded.

Action was slowest post‐delivery for EK horses and fastest for sheep that were not the subject of a PS or EK (Table 5). EK was slower for cattle than for sheep and pigs. Post‐detection decisions of EK were taken fastest, just 0.2–0.7 hour; PS decisions were slower, ranging from 3.7 hours for pigs to 8.3 hours for sheep.

**TABLE 5:** The median times to take action, after delivery and detection, and the time to subsequently communicate the incident, for each Corrective Action in the different species.

Species	Corrective action decision	Delivery–action time (hours)	Detection–action time (hours)	Action–communication time (days)
Cattle	No corrective action	11.7	‐	0
	Priority slaughter	11.0	4.4	5
	Emergency killing	12.0	0.5	1
	Dead on detection/arrival	14.0	0.5	6
Sheep	No corrective action	0	‐	0
	Priority slaughter	11.5	8.3	0.5
	Emergency killing	7.6	0.1	0
	Dead on detection/arrival	6.9	>0.1	0
Pigs	No corrective action	15.4	13.2	1
	Priority slaughter	11.2	3.7	1
	Emergency killing	9.0	0.3	2
	Dead on detection/arrival	14.2	0.2	‐
Horses	No corrective action	‐	‐	‐
	Priority slaughter	‐		
	Emergency killing	15.7	0.7	1
	Dead on detection/arrival	‐	‐	‐

*Note*: ‐ = too little data to estimate time.

### Correlations between the number of incidents, animal conditions, voyage characteristics and mitigation actions

Over all the species, the number of affected animals was greater in larger consignments (Table 6). There were only weak correlations with specific welfare problems, but transport time was longer for larger consignments (CC 0.19). A longer transportation time and a greater number of affected animals correlated positively with an EK decision. Older animals and females also had more EKs. PS was more common if there was a long detection to action time. Animals with handling problems were more likely to be assigned to the NCA category. Long detection to action times were more likely to result in PS and less likely to result in EK.

**TABLE 6:** Correlations matrix with the corrective action, number of animals of each type, animals’ demeanour, animal conditions and voyage characteristics, for all species combined.

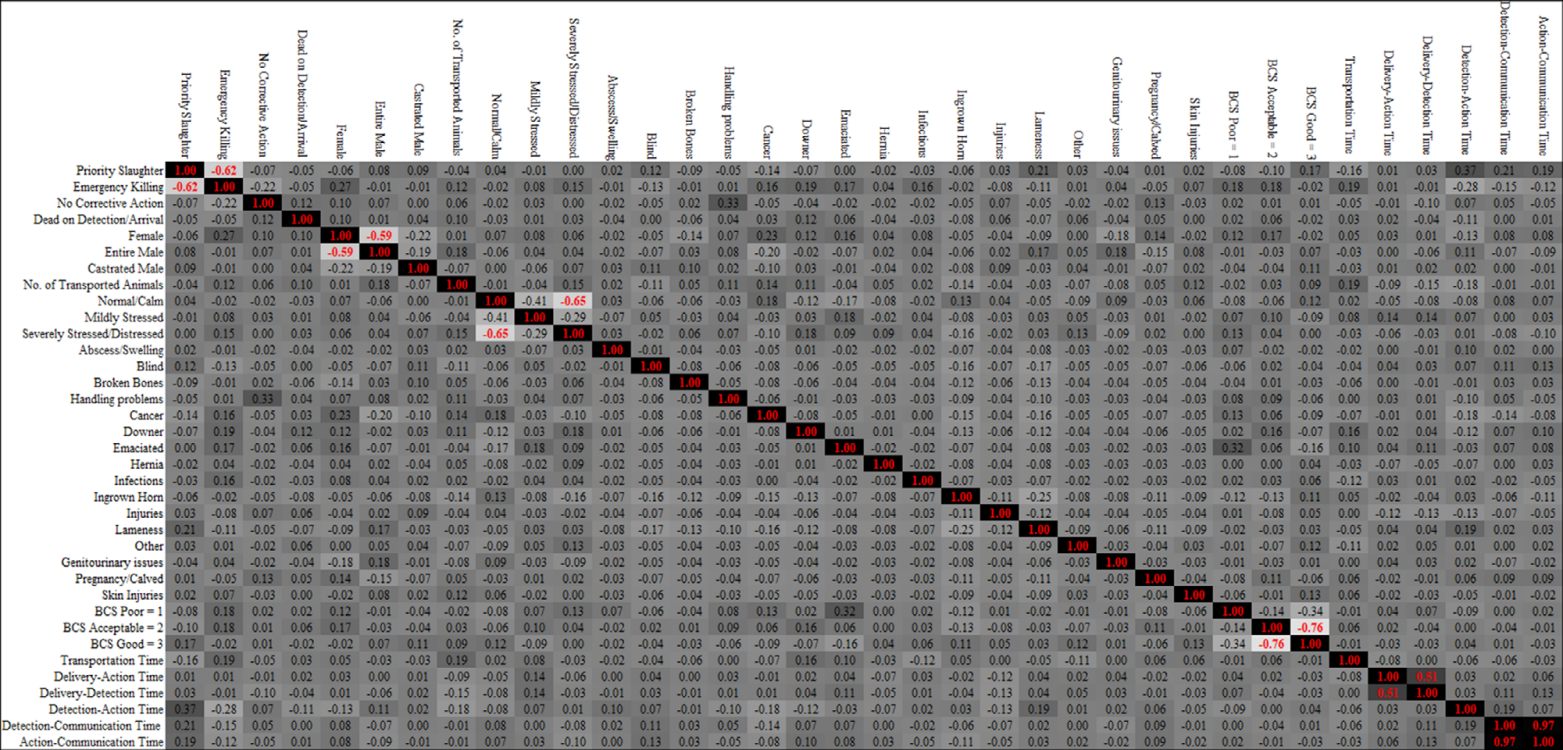

*Note*: Darkest shaded boxes are significant at *p* < 0.001, next darkest shaded at *p* < 0.01, and next darkest shaded at *p* < 0.05, compared with the lightest shade, for which *p *> 0.05.

Older males were more likely to have genitourinary issues, have the highest BCS and be mildly stressed. Older females were more likely to have cancer and be EK.

Separate correlation matrices for cattle, sheep, pigs and horses are available (Box S2 and Box S2.3) and results are summarised below.

### Cattle

For cattle, the selection of the PS option had a moderate negative correlation with age in both females and entire males (*rs* = −0.18; *p* < 0.01 and *rs* = −0.15; *p* < 0.01). Entire males’ age was positively correlated with EK (*rs* = 0.26; *p* = 0.07). The condition with the strongest correlation with the entire male age was genitourinary issues (*rs* = 0.45; *p* < 0.01), and there was a tendency for a correlation with the age of females and pregnancy/calving issues (*rs* = 0.57; *p* = 0.07).

### Sheep

The number of affected individuals/consignment and an EK decision were positively correlated (*rs* = 0.88; *p* < 0.01), as were handling problems and the decision not to kill an individual (*rs* = 0.51; *p* < 0.01). The number of severely stressed animals increased with the number of older neutered males (*rs* = 0.94; *p* < 0.01) so consignments of large numbers of old neutered males were more likely to be reported as severely stressed.

### Pigs

The age of pigs was negatively correlated with BCS score (*rs* = −0.65; *p* = 0.048). As with sheep, handling problems and the decision not to kill were positively correlated (*rs* = 0.99; *p* < 0.01). Infections and skin injuries were the conditions with the strongest correlation with EK (*rs* = 0.48; *p* < 0.01) and PK (*rs* = 0.35; *p* = 0.04), respectively.

### Principal component analysis

**FIGURE 4:**
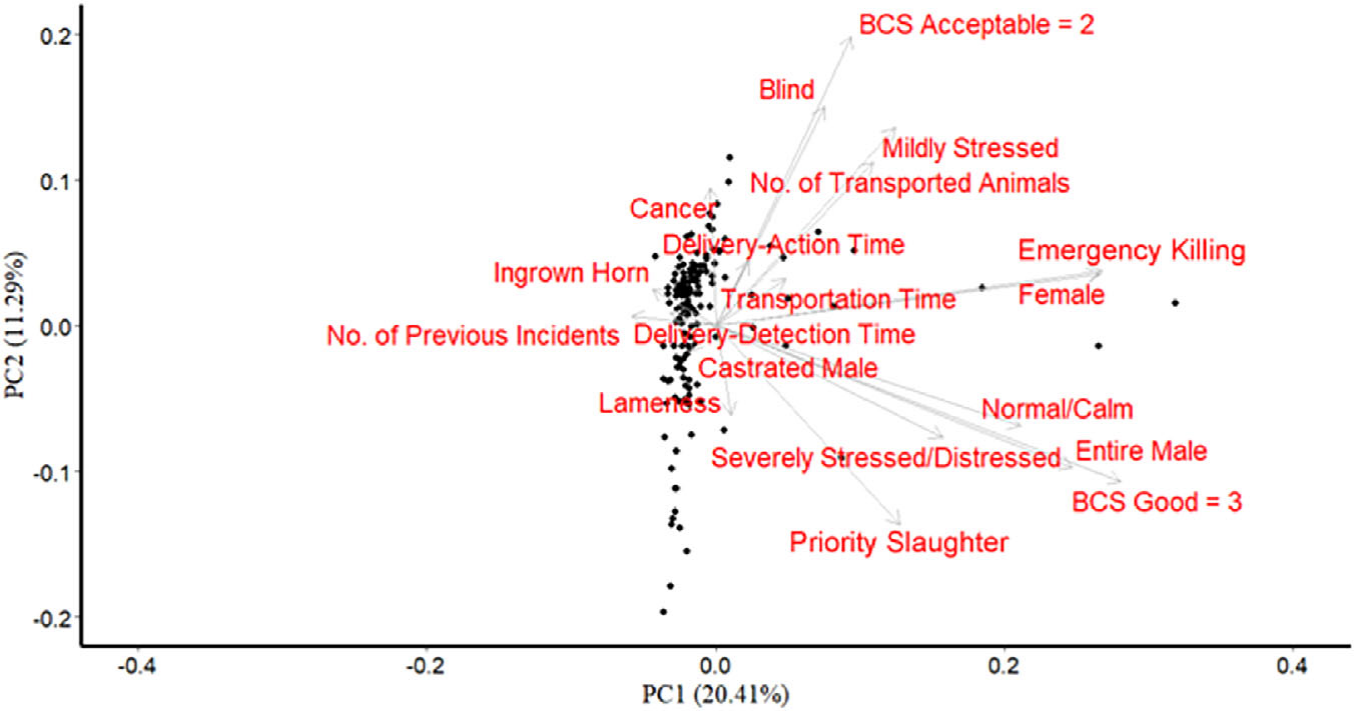
Principal components (PC) identified as PC1 and PC2. The weight (eigenvectors) of variables in the first two principal components.

The first component explained 23.82% of the variation in the dataset. It describes a gradient of records according to the number of affected animals, the number of EK, handling problems, high BCS and normal/calm behaviour. PC2 explained 10.82% of the dataset variation, and it correlated with a gradient of voyages with a higher incidence of blindness in mildly stressed individuals with acceptable BCS (Figure 4). In addition, it was linked with shorter detection‐to‐action, detection‐to‐communication and action‐to‐communication times. PC3 explained 9.7% of data variability and described a gradient of incidents related to blind animals with mildly stressed behaviour and acceptable BCS. PC4 explained 7.4% of the variability and was related to voyages with larger numbers of animals under some level of stress and short delivery‐to‐action times. PC5 was related to PS of distressed animals, explaining 7.1% of the data.

Considering the variables describing the voyage characteristics, the PCA‐loadings indicated that some of these variables were correlated with different corrective actions. For example, the first principal component had a strong association with both the total number of animals affected and assigning an animal for EK. Longer action times were inversely correlated with the implementation of PS. Additionally, voyages with large numbers of animals were correlated with longer delivery‐detection times and fewer animals with normal/calm behaviour.

## DISCUSSION

Even though over twice as many sheep were transported as cattle, there were more incident reports for cattle. This was partly because cattle were more likely to be the subject of an incident (4.6% vs. 3.5% of 25,468), but also because cattle consignments were smaller than sheep consignments. Of the four species, pigs were proportionately most likely to have an incident (11.5% of 7964), and the proportion of the small numbers of horses with incidents (3.8% of 183) was similar to that for sheep (3.5% of 63,885). Cattle were more likely to be recorded as mildly stressed than sheep, and pigs and horses were most likely to be recorded as severely stressed. The small number of horses affected means that caution must be taken in comparing them with other species. As expected, larger consignments had more affected animals, but we did not find that the proportion changed with consignment size. Several interacting factors might be involved here: the longer time large consignments were transported might be expected to have increased numbers affected, but the inspection may not be as rigorous in large consignments, potentially leading to under‐reporting.

Among all the species, the most common condition reported was ‘handling problems’, mostly caused by the driver. We acknowledge that some of the bruising could have been inflicted by another animal, but we have kept the name as this was how the reporting officer classified the welfare problem. Pigs had the highest prevalence, possibly because they are harder to move than the more gregarious sheep and cattle. Handling problems also reflect the quality of the journey, over which the driver has a key influence.^33^ Drivers of farmed animalrive in Europe may be unaware of both the welfare requirements of their animals and the relevant animal welfare laws and regulations.^24^, ^35^ Drivers often blame farmers for sending unfit animals for slaughter,^34^ but legally, at least in the EU, the driver is also responsible for ensuring that animals are fit to travel.^35^ Better training of drivers and collaboration between slaughterhouse workers, farmers and drivers are warranted. The Australian Livestock and Rural Transporters Association (ALRTA) has recently announced that it will revise the accreditation of drivers to improve the animal welfare component.^36^ Almost as common a problem as driver‐handling issues were dog issues. Dogs are frequently used to assist in the movement of both sheep and cattle, and they may lunge towards sheep, stand or run onto their backs, and bark, which distresses the sheep and reduces meat quality.^37^

Sheep were more likely to be reported lame than to have handling problems. In Australia, the high stocking densities, lack of bedding and striations on the floor of the vehicle combine to ensure that animals stand throughout the journey. Fatigue is a significant problem in longer journeys, as animals have to step constantly to maintain their balance,^38^ potentially exacerbating lameness problems. Between 16% (cattle) and 29% (sheep) were classified as lame, but this is likely to be an underestimate as lameness can be difficult to recognise, even for farmers.^39^ In a Canadian survey, the risk of cattle becoming non‐ambulatory, lame or dead increased sharply after 30 hours on a truck, with 30% of cattle transported for over 30 hours dying or becoming non‐ambulatory or lame.^40^ In Australia, some journeys are long because of the extensive nature of farming and the long distances to slaughterhouses. Maximum journey time in Australia is determined by time off the water, which for cattle over 6 months old and sheep over 4 months old is 48 hours.^41^ In all 2,470,000 sheep and cattle were transported from Western Australia to the eastern states between January 2020 and June 2021,^42^ a journey of 30–50 hours. In our study, most animals travelling to the export‐accredited slaughterhouses had been travelling for an average of about 5 hours, but some up to 24 hours. Nevertheless, longer journeys were more likely to have animal incidents that required EK. Our journeys were short on average (Table 4) because although there were some voyages of 24 hours, suppliers to export abattoirs tend to congregate near the slaughterhouse. Sending farmed animals from one side of the country to the other tends not to happen for export.

Pigs, with the highest prevalence of handling problems, were least likely to require EK. For the other species, this was the most likely outcome. Detection of EK was unacceptably long, ranging from 8 to 12 hours for the different species (Table 5). Following detection, the time to take action was rapid, generally less than 1 hour, particularly if EK was required. Hence emergencies are detected earlier than less urgent cases. A further day is required to report the incident, which is long if follow‐up investigations are required. The PCA suggests that there is a gradient from mildly stressed to severely stressed animals, with the latter more likely to be prioritised for slaughter.

The time between arrival and detection of the condition (median 10.8 hours) is a cause for concern. Inspection delays may be caused by animals being delivered late in the day and inspected by the OPV or slaughterhouse staff on the next day, but this still means the affected animals suffer for a considerable period. The decision to kill sheep was taken much quicker than horses, which may be either because of the ease of removing the former for a quick kill or because staff may be authorised to take action for sheep but not horses, for whom they had to wait for a veterinarian's decision.

The decision to kill an animal in situ requires careful consideration. These animals must be experiencing considerable pain, unable to walk to the kill floor and in urgent need of slaughter. Sight and/or sound and/or scent of the killing of a conspecific may cause distress to other farmed animals in the vicinity,^43^ although the evidence for this is equivocal and differs between farmed animal species. Olfactory cues from stressed cows can make heifers exposed to those cues fearful,^44^ confirming earlier work on rats.^45^ During slaughter, cattle postural changes in response to stressful events may be interpreted by, and induce fear in, conspecifics.^46^ Pigs have shown responses to sounds that are indicative of distress, increasing their responses with the intensity and frequency of the sounds, but not in response to conspecific vocalisations.^47^ If the suffering is too great, it is likely that emergency slaughter will be the better course of action, regardless of stress caused to other animals.

The main limitation of this study was the relatively short time period of data collection. Although there were many reports for cattle, sheep and pigs, there were few for horses, from which limited conclusions could be drawn. The replication of other species that we investigated was sufficiently large (in the thousands) to ensure that the sample size did not affect the statistical output. Animals’ age was not included in the PCA due to the low variance of observations.

Some of the welfare problems are likely to have occurred because animals were loaded that were not fit to travel, hence action should be taken to ensure greater enforcement of the requirements for animals to be fit to load. Many of the issues reported were long‐term problems, in particular abscesses, cancers, ingrown horns, lameness and genitourinary issues and could have been detected before the journey. Further, the suffering of animals delivered to slaughterhousescould be reduced by ensuring prompt inspection and immediate action upon delivery at the slaughterhouses to either treat or kill unfit animals. We have developed a list of measures and actions to improve welfare‐related outcomes in the future (Table 7).

**TABLE 7:** List of measures and actions to reduce non‐compliance.

Action	Reason
Improve farm inspections	To determine those who are not fit to sell and transport to a slaughterhouse, and treat or humanely kill those animals
Improved training in the assessment of animal welfare	To detect animals unfit to sell or transport
Remove payment	Remove payment made by slaughter facilities for unfit animals to vendors, as payment incentivises the trade
Review transit insurance	Transit insurance also incentivises the transport of unfit animals
Review National Vendor Declaration	The fact that a declaration is made if the vendor is supplying suspect/lame/injured animals suggests to vendors that they are permitted to transport unfit animals and so they do not necessarily think that it may breach relevant legislation
Add more information on saleyards and the origin of animals	Slaughter plant buyers buy at various saleyards and the animals may have crossed State or Territory borders, so they must be aware that they might be buying unfit animals
Real‐time data	Management controls for transport journeys so that real‐time transport times are relayed to a central collection point, and from there to regulators
Improve legislation for animal transportation	Better handling of animals by some transporters needs to be addressed in legislation
Reduce time period extension for time off water and feed	Various time periods of time off water and feed, e.g., in one report animals were off feed for 67 hours
Improve animal inspection	Gaps between the time of delivery to the slaughter plant and the time that the animal was detected because of no receiving staff working outside production hours (mostly sheep but occurs in some cattle yards), failure to detect and correctly assess AWI, and lack of training the staff responsible do not have the authority to destroy the animal or call someone in who does have the authority Significant time delays between the time of delivery, detection and taking action Reliance on the OPV to identify animals and to decide what action should be taken, rather than the slaughterhouse having a robust system that would ensure animals who are welfare compromised are identified and attended to without delay Training of the staff responsible for welfare needs to be reviewed, to ensure a comprehensive understanding of conditions that are painful and how they should go about detecting these painful conditions. Although the AWIR acknowledges the condition as being one that causes pain, their actions do not always match the appropriate response required in dealing with the animal
Change AWIR format	The AWIR is paper‐based rather than electronic. Some OPVs are unwilling to devote the time to completing the reports and some report that many animals with conditions are not reported
Change AWIR provision to authorities	There needs to be provision of AWIR to State authorities in a timely manner
Improve inspection on‐farm	Proactive regulatory responses to inspection of on‐farm practices. Changes in legislation may be required to allow on‐farm inspections
Implement OFES service (on‐farm emergency slaughter)	Lack of service and economic benefit
Change transportation payments	Changing the transportation payments based on the condition of animals at delivery, not paying the drivers on the basis of kilograms of livestock transported, to avoid the overloading of animals and providing rewards to the slaughterhouse if there is a reduction of bruising and dead animals arriving at the slaughterhouse

## AUTHOR CONTRIBUTIONS

All authors made substantial contributions to the conception and design, acquisition of data, and/or analysis and interpretation of data. Francesca Carnovale and Dawn Lowe collated and analysed the data. All authors drafted the manuscript and/or revised it critically for important intellectual content. All authors agreed to be accountable for all aspects of the work and ensured that questions related to the accuracy or integrity of any part of the work were appropriately investigated and resolved.

## CONFLICTS OF INTEREST

The authors declare they have no conflicts of interest.

## FUNDING INFORMATION

The authors received no specific funding for this work.

## ETHICS STATEMENT

Ethical approval was not required because the research involved information freely available in the public domain.

## Data Availability

The raw data have not been published or stored elsewhere but are available on request from Francesca Carnovale or Dawn Lowe.
